# Book Review: Unwritten Rules of Social Relationships: Decoding Social Mysteries Through Autism's Unique Perspective

**DOI:** 10.3389/fpsyg.2020.615151

**Published:** 2020-11-04

**Authors:** Vanita Sharma, Rashmi Gupta

**Affiliations:** Cognitive and Behavioural Neuroscience Laboratory, Department of Humanities and Social Sciences, Indian Institute of Technology Bombay, Mumbai, India

**Keywords:** autism, emotion, cognition, non-verbal cues, social interaction

Autism Spectrum disorder is characterized by persistent difficulties in communication and social interactions across multiple contexts [DSM-V, (American Psychiatric Association APA, 2013)]. They show restricted and repetitive behaviors. A first-hand information approach helps one to have a better understanding of the experiences and workings of a mind with autism. Dr. Temple Grandlin and Sean Barron, who have autism, attempted this through a book titled “Unwritten Rules of Social Relationships.” Dr. Grandlin is a university professor and accomplished author while Sean Barron is a well-established newspaper reporter.

The book is divided into three parts. In the first part, the authors discussed how their professional life experiences helped them to deal with autism-related issues. Temple follows a methodical logic way of life to navigate her road through Autism; Sean has an emotion infused approach for it. During adolescence, Temple had trouble interacting with her peers. However, her work experience helped her to develop good work ethics and be a responsible person. Due to repetitive behavior tendency and poor emotion recognition and regulation, Sean had difficulty to interact with peers and family (Samson et al., 2013). Positive support from his family and being a journalist enabled him to deal with his autism-related issues.

The second part of the book gives an insight into the thinking pattern of individuals with Autism and how it impacts their social life. It has been highlighted that rather than looking at the bigger picture, they pay attention to the minute details of the environment (Behermann et al., 2006). Temple compared the mind of individuals with autism with Google search engine and suggested that an autistic mind sifts through a lot of details before arriving at a relevant one (Johnson et al., 2010) when things are imprecise, which explains why individuals with autism take longer to respond (Baisch et al., 2017).

In the third part of the book, the authors proposed 10 explicit guidelines (rules) with practical suggestions to caregivers that could help individuals with autism to improve their personal and social life. Healthy individuals can easily understand these guidelines. However, often individuals with Autism fail to learn or understand these guidelines unless these are explicit to them. Social interactions consist of unwritten guidelines that vary based on the context and with different groups of people. For example, Sean was taught to tell the truth always. However, in some circumstances telling the truth can hurt the feelings of others. This is illustrated in the book with a personal example of Sean where his reply (“I hate it”) to the host regarding the quality of food served showed a lack of understanding of social conventions. In the first guideline, authors provided a practical suggestion to caregivers by refraining to use words like “*always”* or “*never.”* Instead, they suggested using the phrase “*in this situation”* to improve their social interactions.

Individuals with Autism give equal weightage to each piece of information irrespective of its order of importance; therefore, they are unable to categorize the intensity of felt emotion driven by a different context. For example, individuals with autism react (e.g., anger outbursts) to the situation irrespective of whether the situation is big (e.g., getting fired from the job) or small (e.g., minor disagreement with a family member). In the second guideline, caregivers are suggested to use concrete strategies (e.g., feeling charts with scales, colors, or numbering systems) to teach them that not everything is equally important in the grand scheme of things.

Individuals with autism view a small mistake (e.g., putting some pieces wrongly in a puzzle) as complete failures giving rise to self-inflicted stress and anxiety. In the third guideline, it is recommended that caregivers help them understand that mistakes are related to what they do or say and not who they are.

Individuals with autism follow honesty to an absolute degree and that it can also affect others' feelings. This is illustrated in the book with a personal example of Sean where he shouted “I already have this,” in front of all his guests, about a gift that he received from his friend at his birthday party. In the fourth guideline, caregivers are advised to teach them the difference between situations that require honesty to situations that require diplomacy.

In the fifth guideline, caregivers are advised to pay attention to the development of basic social functioning skills (e.g., polite behavior) in individuals with Autism. Being polite would also help them to relate with others emotionally.

Individuals with Autism have difficulty understanding the hidden motives or emotions of other people. In the sixth guideline, caregivers are suggested to teach them skills such as paying attention to non-verbal visual cues (e.g., facial expressions, body-posture, eye contact, etc.) to decode bad intentions (e.g., sexual assault) of others. These skills would help them to recognize deception, which is an important skill for safety.

Individuals with autism often fail to understand social norms/contextual information (e.g., people behave differently in public than in private settings). In the seventh guideline, caregivers are advised to teach individuals with Autism to pay attention to intangible clues to understand the contextual information better. Similarly, due to poor understanding of non-verbal communication, individuals with autism are unable to recognize that the other person has lost interest in an on-going social interaction. Some factors that give rise to disinterest in social interactions are poor hygiene, invading one's personal space, and not letting anyone else talk. In the eighth and ninth guidelines, caregivers are suggested to train them using visual tools (e.g., turn-taking cards, video-recording of autistic behavior) and context-appropriate clothing for better social interactions. Authors also suggested caregivers engage individuals with Autism in small talk (e.g., greeting and complimenting peers) to express themselves within socially accepted boundaries.

In the last guideline, authors suggested caregivers educate individuals with autism that appropriate behavior in a social setting is a lifelong learning process that requires personal effort and individuals are responsible for their behavior. However, authors further raise awareness in caregivers to acknowledge the fact that all troubles in social interactions are not an individual with autism's fault. Caregivers are also encouraged to use positive visual and verbal reinforcement (e.g., praising them for good behavior), and teaching them better emotional regulation strategy (e.g., replacing negative self-talk with positive self-talk) for behavior modification.

In conclusion, this book provides an incredible insight into the world of a person with autism. The guidelines written in the book are backed up with authors' powerful stories. These guidelines could help caregivers to make the personal and social life of individuals with autism better. Authors encourage individuals with autism to focus on developing their talents and translating it to suitable professions, thereby living independent and fulfilling lives. The current need is to have a better understanding of the cognitive and emotional mechanisms in children and individuals with autism. It will aid the development of intervention tools that will help them deal effectively in social settings.

## Author Contributions

VS and RG wrote the paper. All authors contributed to the article and approved the submitted version.

## Conflict of Interest

The authors declare that the research was conducted in the absence of any commercial or financial relationships that could be construed as a potential conflict of interest.
